# Potential Role of Nuclear Factor *κ*B in Diabetic Cardiomyopathy

**DOI:** 10.1155/2011/652097

**Published:** 2011-05-03

**Authors:** O. Lorenzo, B. Picatoste, S. Ares-Carrasco, E. Ramírez, J. Egido, J. Tuñón

**Affiliations:** ^1^Vascular Pathology Laboratory, Instituto de Investigaciones Sanitarias IIS-Fundación Jiménez Díaz, Av. Reyes Católicos 2, Madrid 28040, Spain; ^2^Cardiology Department, Instituto de Investigaciones Sanitarias IIS-Fundación Jiménez Díaz, Av. Reyes Católicos 2, Madrid 28040, Spain

## Abstract

Diabetic cardiomyopathy entails the cardiac injury induced by diabetes independently of any vascular disease or hypertension. Some transcription factors have been proposed to control the gene program involved in the setting and development of related processes. Nuclear factor-kappa B is a pleiotropic transcription factor associated to the regulation of many heart diseases. However, the nuclear factor-kappa B role in diabetic cardiomyopathy is under investigation. In this paper, we review the nuclear factor-kappa B pathway and its role in several processes that have been linked to diabetic cardiomyopathy, such as oxidative stress, inflammation, endothelial dysfunction, fibrosis, hypertrophy and apoptosis.

## 1. Introduction

Cardiac diseases are the primary cause of death worldwide and their associated pathologies have their origins in alterations of gene expression. Though many causes of cardiac injury have been identified, the key molecular mechanisms of their pathogenesis have not been understood. The molecular processes within the cardiac cell are controlled by transcription factors such as nuclear factor-kappa B (NF-*κ*B). In many heart diseases there is an increased NF-*κ*B activity. In particular, NF-*κ*B activation is involved in congestive heart failure, myocardial hypertrophy, diabetic cardiomyopathy (DC) and coronary artery disease, including acute coronary syndromes and ischemic reperfusion. In this paper we will focus on the potential role of NF-*κ*B activation in DC. This entity includes the direct effect of diabetes mellitus, as one of the most prevalent diseases, on the myocardium. NF-*κ*B activation may act in DC via canonical and non-canonical pathways, by controlling different set of genes and being involved in related processes such as oxidative stress, inflammation, endothelial dysfunction, fibrosis, hypertrophy and apoptosis.

## 2. Nuclear Factor-***κ***B Signaling

NF-*κ*B is a ubiquitous family of inducible dimeric homodimer or heterodimer transcription factors including five members: Rel (c-Rel), RelA (p65), RelB, NF-*κ*B1(p50/p105) and NF-*κ*B2(p52/p100). All of these proteins share a highly conserved Rel homology region of about 300 amino acids, composed of two immunoglobulin-like domains [1–4]. This region is responsible for the interaction with other members of the family, inhibitory proteins and DNA. In non-stimulated cells, homodimers or heterodimers of NF-*κ*B family members are bound to ankyrin-rich regions of NF-*κ*B inhibitory proteins (I*κ*B). Thus, the NF-*κ*B-nuclear localization sequences are masked, and NF-*κ*B remains sequestered in the cytoplasm [2, 3]. Among the different tissues, two principal signaling cascades, the classical/canonical NF-*κ*B activation and the alternative/non-canonical pathway, can control NF-*κ*B activation and nuclear transport in the heart. 

**Figure 1:**
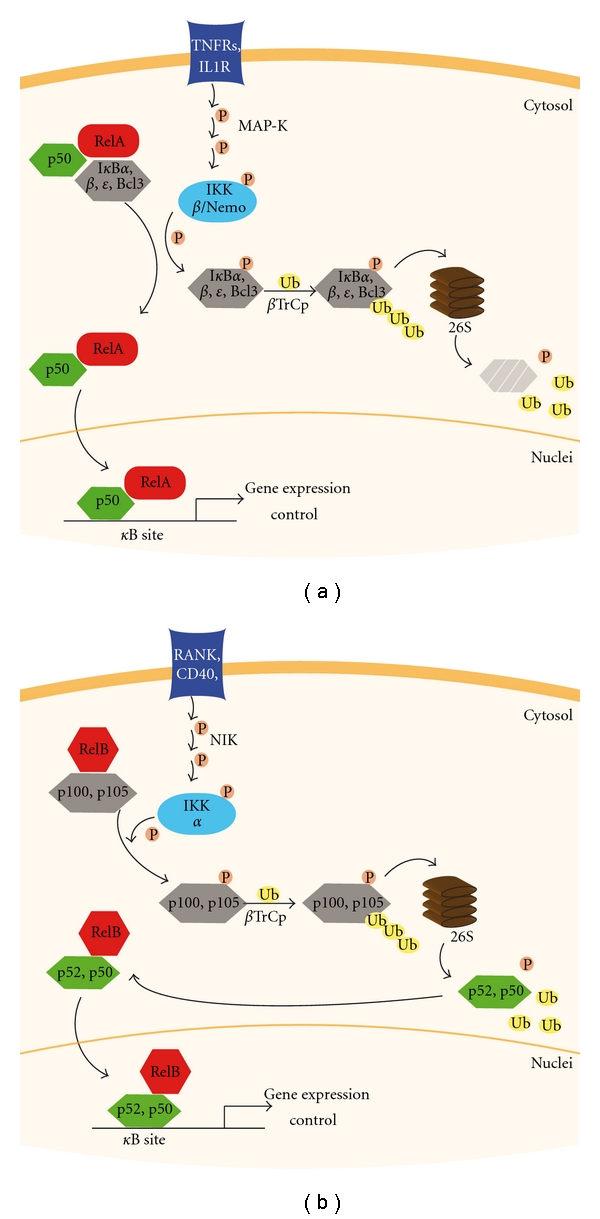
Canonical and non-canonical NF-*κ*B signalling pathways. (a) The classical NF-*κ*B pathway activation follows a trans-phosphorylation chain from the specific activated receptors to the I*κ*B proteins. This triggers NF-*κ*B heterodimer to entry into the nucleus and control the gene expression by binding to *κ*B sites within promoter regions. (b) In the non-canonical signaling, trans-phosphorylation and proteasome degradation are needed before active NF-*κ*B can access into the nucleus. TNFR: TNF*α* receptor; IL1R: IL-1*β* receptor.

The canonical pathway proceeds via phosphorylation, ubiquitination, and proteasomal degradation of specific I*κ*B, in particular I*κ*B*α*, I*κ*B*β*, I*κ*B*ε* or Bcl-3 (Figure 1(a)). The phosphorylation occurs at two serines (S32 and S36) in the aminoterminus of I*κ*B and is catalyzed by I*κ*B kinases (IKKs)-*α* and mostly *β*, complexed with the regulatory subunit NEMO (NF-*κ*B essential modulator). Phosphorylation promotes lysine 48- (K48-) linked polyubiquitination at adjacent lysine residues initiated by the ubiquitin E3 ligase complex SCF^*β*TrCp^ [3]. Ubiquitination leads to proteolysis of the NF-*κ*B-bound I*κ*B at the 26S proteasome. Thus, free ubiquitin can be reused for I*κ*B degradation signaling. Free NF-*κ*B dimers, commonly the RelA/NF-*κ*B1(p50) heterodimer, translocate into the nucleus through interaction with karyopherins (nuclei-cytoplasmic transporters). Activated NF-*κ*B bind NF-*κ*B-DNA sites (named *κ*B sites) to regulate gene transcription. Interestingly, knockout mice for all of the NF-*κ*B subunits have been obtained, but only the RelA knockout is non-viable, suggesting some functional redundancy among other members of the family [4]. 

The non-canonical route starts through the phosphorylation, ubiquitination, and proteasomal degradation of inhibitory NF-*κ*B1(p105) and NF-*κ*B2/p100 proteins (Figure 1(b)). This pathway is much slower and weaker than the former and depends on IKK*α* but not IKK*β* or NEMO. NF-*κ*B1(p105) and NF-*κ*B2/p100 are phosphorylated at their carboxyl-terminus and then K48-polyubiquitinated by SCF^*β*TrCp^ ligase [4]. Subsequent proteasomal processing of NF-*κ*B1(p105) or NF-*κ*B2/p100 generates mature p50 or p52, respectively, which heterodimerizes with RelB. Nuclear translocation of these heterodimers and transcriptional activation of distinct target genes follow. 

IKK complexes appear to be the most likely point of convergence of many NF-*κ*B regulators. Some NF-*κ*B inhibitors target IKKs competing for their ATP binding site, abrogating I*κ*B phosphorylation and degradation, and thereby NF-*κ*B activation. In this sense, deubiquitinating enzymes such as CYLD and A20 can also block NF-*κ*B activation by removal of ubiquitinated chains [4, 5]. IKK can even directly phosphorylate RelA subunit, which increases its transactivation strength [2, 4]. In addition, IKK-phosphorylated targets, I*κ*Bs, play critical roles in the regulation of NF-*κ*B [6]. As an example, the absence of IKB*α* in knockout mice produces a lethal increase of skin inflammation and proinflammatory molecules [7].

## 3. NF-*κ*B-Regulated Genes

NF-*κ*B was initially identified as a nuclear factor bound to the enhancer of the immunoglobulin-*κ* light chain gene of B-lymphocytes. However, it is clear that several dozens of NF-*κ*B : I*κ*B complexes may exist within the cells, providing an enormous diversity of options and allowing for specific regulations in the NF-*κ*B system. Thus, different activated NF-*κ*B dimers bind with distinct affinity to a variety of consensus *κ*B sites in the heart [8]. The *κ*B sites [5′-GGG(A/G)NN(C/T)(C/T)CC-3′; where N is any base] can be uneven resulting in subtle cell and gene-specific modulations of gene expression. Importantly, NF-*κ*B dimers do not promote gene transcription by themselves, but as a part of a complex of several coactivators such as the cAMP Response Element Binding Protein or Signal Transducers and Adaptors of Transcription [8, 9]. Moreover, NF-*κ*B interacts with a variety of other transcription factors in a positive or negative manner. In some cases, like Nuclear Factor-IL-6 and activator protein-1 (AP-1), the interaction results in a synergistic stimulation of inflammatory genes [1, 3, 10]. On the contrary, the physical association of NF-*κ*B and the glucocorticoid receptor (another transcription factor) inhibits NF-*κ*B DNA binding (and enhances I*κ*B*α* levels) [11]. Peroxisome proliferator activated receptor (PPAR) is another transcription factor able to avoid the NF-*κ*B nuclear translocation [12].

NF-*κ*B controls the expression of some 200 target genes. Many of them are involved in inflammation, such as adhesion molecules, interleukins, chemokines, acute phase response genes and cytokines [2, 6, 8]. Some of these cytokines are TNF-*α* and IL-1*β*, closing the circle of a self-sustained loop (Table 1). Also NF-*κ*B regulates a large number of immune responses, stress, surface receptors, cell survival, growth factors, proliferation and ubiquitin-proteosome system genes. Interestingly, the RelB, c-Rel, NF-*κ*B1(p105) and I*κ*B*α* gene expression is under NF-*κ*B control [9].

## 4. NF-*κ*B and Diabetic Cardiomyopathy

Type-I and type-II diabetic patients are at increased risk of cardiomyopathy and heart failure is a major cause of death for these patients [13–15]. Two-thirds of the diabetic patients die because of cardiovascular complications [16]. In the Framingham heart study, the risk of heart failure was increased in diabetic men and women by two- and fivefold, respectively [17]. This could not be explained by looking at several other diabetes mellitus-associated risk factors such as obesity, dyslipidemia, infarction, autonomic neuropathy, or endothelial dysfunction. Thus, diabetes mellitus alters cardiac structure and function independently of coronary artery disease and systemic hypertension, a condition known as diabetic cardiomyopathy (DC) [18]. Here, the associated hyperglycemia and hyperlipidemia induce severe cardiac alterations (metabolic and structural) that lead to ventricular diastolic and systolic dysfunction, typically a delayed relaxation pattern and presence of left atrial enlargement [19]. However, there is an uncompleted knowledge of the DC-associated cellular mechanisms [18], including NF-*κ*B signalling.

**Figure 2:**
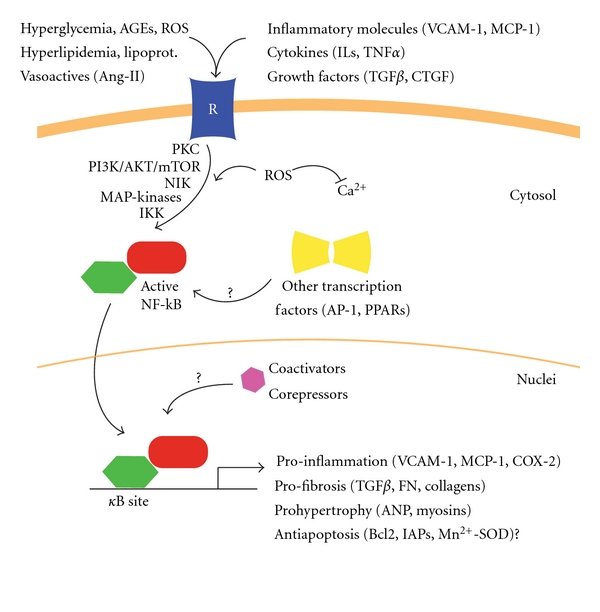
NF-*κ*B and DC-associated gen expression. Under diabetes mellitus, hyperglycemia, hyperlipidemia and other stimulated factors, such as cytokines or inflammatory molecules, activate the NF-*κ*B pathway in the heart. NF-*κ*B, depending on other transcription factors and coactivators/repressors interactions may regulate the expression of involved genes via different mediators. CTGF: connective tissue growth factor; ILs: interleukins; FN: fibronectin.

NF-*κ*B can be activated by a large number of both physiological and non-physiological stimuli, including cytokines, mitogens, viruses, mechanical and oxidative stress (Table 1). However, at present it is unclear how various intracellular and extracellular stimuli converge to trigger NF-*κ*B activation. Myocardial tissue from patients with heart failure of various etiologies exhibits activation of NF-*κ*B and overexpression of NF-*κ*B-regulated genes [20]. In DC, the high concentrations of circulating glucose and LDL/VLDL lipoproteins stimulate myocardial NF-*κ*B activation [21, 22]. Also they lead to the release of vasoactive peptides (i.e., angiotensin-II, endothelin-1, phenylephrine) and growth factors (TGF*β*, Connective Tissue Growth Factor) from the circulating and local cells [23–26]. These molecules could stimulate NF-*κ*B activity directly or by cytokine-mediated expression (Figure 2). In the canonical pathway, under potent stimulus such as cytokines (TNF*α*, IL-1*β*) or lipopolysaccharide, I*κ*B*α* is rapidly degraded within minutes [9]. TNF*α* and IL-1*β* activate NF-*κ*B primarily through interaction with their respective type-1 receptors, which bind to adapter molecules such as TNF Receptor-Associated Factors. Then, these adapters can interact with mitogen-activated protein- (MAP-) kinase, which transphosphorylate and activate IKK [8]. In the non-canonical pathway, cellular receptors such as receptor activator of NF-*κ*B, CD40 and lymphotoxin-*β*R stimulate NF-*κ*B [3]. Other stimuli like double-stranded RNA result also in NF-*κ*B activation [27]. In these cases, a common interacting protein (NF-*κ*B Inducing Kinase) can transphosphorylate to I*κ*B proteins [28]. 

Most information on pathogenic mechanisms in DC is derived from animal models [29, 30]. Some *in vivo *and *in vitro *data suggest the NF-*κ*B participation. An increase of NF-*κ*B activity in a diabetic [10] or diabetic-hypertensive [31] rat heart has been described. In cultured cardiomyocytes, incubation with high-glucose promotes NF-*κ*B activation through the diacylglycerol-PKC signal transduction pathway. This route increased PKC*α* and PKC*β*2, which phosphorylated MAP-kinase and lately I*κ*B [22]. The cardiac responses to DC are oxidative stress, inflammation, endothelial dysfunction, fibrosis, hypertrophy and apoptosis, in which NF-*κ*B could participate.

### 4.1. NF-*κ*B and DC-Associated Oxidative Stress

Hyperglycemia and subsequently generation of advanced glycation end-products (AGEs) are major promoting sources of reactive oxygen species (ROS) within the cardiomyocytes [32] (Figure 2). Another cause of ROS accumulation in the cardiomyocyte can be the excess of mitochondrial oxidation, mainly from fatty acid degradation (almost the unique origin of fuel in DC) [33]. Some ROS, as superoxide, react with nitric oxide lacking its beneficial signalling action, or are converted to hydroxyl radical, which damages proteins, lipids and DNA. The elevation of free radicals (and AGEs) also impairs Ca^2+^ translocation (by Ca^2+^-pump decrease) and triggers NF-*κ*B activation, reducing cardiac contractibility and modifying the overall gene expression program in the diabetic cardiomyocytes [23, 33]. In particular, PKC-*α* and PKC-*β*2 overexpression may be mediating the NF-*κ*B activation induced by high content of ROS [34]. Importantly, as well as hyperglycemia, cytokine stimulation (TNF*α*, IL-1*β*) may activate NF-*κ*B via ROS formation [1, 23]. Thus, oxidative stress will become a common mediator involved in the initiation and development of the cardiac responses to DC.

### 4.2. NF-*κ*B and DC-Related Inflammation

The inflammatory response is a significant process in the progression of heart failure in DC. Although cardiac inflammation is not present in chronic experimental models of diabetes mellitus (by the local expression of anti-inflammatory and anti-oxidant molecules), it has been demonstrated as an early response to the diabetic insult [35, 36]. In human myocardial biopsies or necropsies there is a lack of information showing local inflammatory leukocytes [37]. However, non-specific serum inflammatory markers such as MCP-1, IL-6, TNF*α*, troponin or C-reactive protein, have been linked to cardiovascular dysfunction in diabetic patients [38, 39]. 

NF-*κ*B is clearly one of the most important regulators of pro-inflammatory gene expression. Cardiac cells express receptors for pro-inflammatory cytokines, IL-1*β* and TNF*α* and also contain the IKK complex needed for NF-*κ*B signal activation [6, 8]. Interestingly, I*κ*B*α* knockout mice are born normally, but usually die a few days after birth because of extensive skin inflammation consistent with persistent NF-*κ*B activation [40]. In early experimental models of DC, activation of NF-*κ*B was correlated with an increased of IL-1*β* and TNF*α* [36, 41]. Thus, NF-*κ*B could lead to or enhance expression of host genes, including cytokines, leukocyte adhesion molecules, cyclooxygenase-2 and inducible nitric oxide synthase, ending to a positive feedback loop (Figure 2). In addition, pro-inflammatory cytokines produced by macrophages, T-cells and cardiac cells may exert their actions on cardiomyocytes and fibroblasts via NF-*κ*B activation [23, 40].

Although inflammation in human cardiac tissue has not been entirely demonstrated, animal studies indicate that the presence of leukocytes in the diabetic heart and NF-*κ*B activation could play a crucial control. Accordingly, most of the beneficial effects of DC treatments improve inflammatory events and NF-*κ*B alleviation (see later).

### 4.3. NF-*κ*B and DC-Involved Endothelial Dysfunction

Endothelial dysfunction is primary cause of atherogenesis initiation in the coronary vasculature that enforces the direct deleterious effect of diabetes mellitus on the heart. Inflammation plays a central process in the atherosclerosis plaque formation [42]. Elevation of circulating glucose, ROS, lipoproteins and cholesterol damages the endothelial cell layer increasing the attraction and adhesion/rolling/extravasation of inflammatory cells. Glycosylated lipoproteins may also serve as inflammatory focus. Cytokines (IL-6, TNF*α*), angiotensin-II and adhesion molecules (ICAM-1, VCAM-1, E-selectin) are released from endothelial cells. As a result, more leukocytes migrate to subendothelial sites of inflammation [42]. Once there, they promote vascular smooth muscle cells attraction (by chemokines MCP-1 and IL-8) from the media layer and fibrosis (by collagen deposition), leading to atherosclerotic plaque formation. The regulation of all these factors by NF-*κ*B has been well described in human and animal models [42]. Finally, the stability of the plaque and thrombosis can depend on the ECM turnover and presence of pro-thrombotic factors. In this sense, as well as MMPs, NF-*κ*B also regulates pro-thrombotic genes.

### 4.4. NF-*κ*B and DC-Linked Fibrosis

In the heart, vasoactive peptides and growth factors (angiotensin-II, TGF*β*) released in diabetes mellitus can activate NF-*κ*B [10]. TGF*β* stimulates the synthesis of extracellular matrix (ECM) proteins including collagens, fibronectin, tenascin and proteoglycans [43] (Figure 2). Interestingly, a close relationship between TGF*β* and NF-*κ*B signaling has been suggested and NF-*κ*B can also interact with AP-1 to overexpress collagens, fibronectin and TGF*β*, enhancing the ECM accumulation [10, 44].

Cardiac injury after DC may result in acute loss of a large number of myocardial cells leading to the formation of a scar. In human biopsies, interstitial and perivascular fibrosis were present in the diabetic heart [37]. In short- and long-term diabetic models, fibrosis and fibronectin expression were augmented [35, 36, 41]. Fibronectin is a major component of the ECM. This glycoprotein possess in its promoter region a specific sequence to be regulated by NF-*κ*B DNA binding. Other NF-*κ*B-induced ECM proteins are elastins [45]. Nevertheless, fibrotic deposition depends on the balance between ECM proteins and ECM-degradation enzymes. Interstitial metalloproteases (MMPs) have the ability to completely degrade collagen and most other ECM components. TGF*β* suppresses matrix degradation through down-regulation of proteinases, such as plasminogen activators and collagenases (i.e., MMPs), and increases synthesis of proteinase inhibitors, such as Plasminogen Activator Inhibitor-1 and tissue inhibitors of metalloproteases (TIMPs). However, paradoxically at least MMP-1, 3, and 9 can be also up-regulated by NF-*κ*B [46], and TIMPs are either up or down-regulated by NF-*κ*B activity [47, 48]. Nevertheless the balance of pro-over anti-fibrotic factors will promote ECM deposition in DC [29, 30]. The up-regulation of profibrotic cytokines and deposition of ECM proteins (via NF-*κ*B, among others) impair contractibility of cardiomyocytes and lead to cardiac stiffness and heart failure progression in DC.

### 4.5. NF-*κ*B and DC-Induced Hypertrophy

NF-*κ*B and its relationship with cardiac hypertrophy are an exciting area of research. In other hypertrophic cardiomyopathy such as dilated cardiomyopathy, there was not genotype or allelic association between a promoter polymorphism [linked to inactivation of NF-*κ*B1(p50/p105)] and the occurrence of heart failure. However, this polymorphism may worsen the onset of the disease by promoting cardiac remodeling [49]. In DC, as initial compensatory response to stress (glucose osmolarity, ROS oxidation), cardiomyocytes increase cellular size through stimulation of contractile proteins like myosins and overexpression of embryonic genes such as the Atrial Natriuretic Peptide [50]. We previously demonstrated also an increase of cell size and Atrial Natriuretic Peptide expression in the rat diabetic heart [35]. 

NF-*κ*B activation can be necessary and sufficient to induce myocardial hypertrophy via G-Proteins stimulation [51] or toll-like receptors (TLRs) [52]. The later mediate their cellular responses by PI3K/Akt/mTOR signaling and stimulation of NF*κ*B (Figure 2). In particular, TLR4 up-regulation has been observed in human heart failure and ischemic hearts, and TLR4-deficient mice showed reduced cardiac hypertrophy following pressure overload [52]. Another potential mechanism involved in the cardiac prohypertrophic NF*κ*B-dependent pathway includes the MAP-kinases/PPAR activation. Cardiac hypertrophy induced by angiotensin-II (via MAP-kinases) was attenuated in p50 null mice [53]. In concordance, TNF*α*-overexpressing mice with cardiac hypertrophy and TNF*α*-induced hypertrophic cardiomyocytes showed reduction of PPAR*γ* coactivator-1*α* expression and its targets (PPAR isoforms). Interestingly, by NF-*κ*B inhibition or incubation with a specific inhibitor of p38-MAP-kinase, cardiac hypertrophy and glucose-induced oxidation were reverted [54, 55]. Thus, the shift in glucose metabolism to fatty acid degradation during cardiac hypertrophy may be also improved by MAP-kinase and NF-*κ*B blockade (or PPAR activation). In consonance, we have preliminary found hypertrophy inhibition in glucose-treated cardiomyocytes after PPAR*β*/*δ* agonist incubation (unpublished data). Of note, mice lacking the NF-*κ*B Interacting Protein-1, a repressor of NF-*κ*B, showed a severe and rapidly progressive dilated cardiomyopathy but not ventricular hypertrophy [56].

Cardiac remodeling might function as an adaptive mechanism to replace dead cells and maintain the heart contractile function. However, hypertrophy can be a *bona fide* apoptosis trigger since the increase of cytoskeleton proteins is a major cause of cell stiffness, contractile dysfunction and death [50].

### 4.6. NF-*κ*B and DC-Associated Apoptosis

Cardiomyocytes rarely proliferate within the adult heart, and cardiomyocyte loss plays an essential role in both animal and human DC [57, 58]. NF-*κ*B signalling can increase the pro-apoptosis gene expression depending on the stimuli and cellular context. In particular, NF-*κ*B is required for endoplasmic reticulum stress-mediated apoptosis [1, 59]. However, NF-*κ*B is usually cytoprotective. The anti-apoptotic effect of NF-*κ*B is very likely to be induced by TNF*α*, although the genes involved in this action are not well characterized [60]. Another postulated effector is cardiotrophin-1. This IL-6 family member activates NF-*κ*B through MAP-kinases and Akt to mediate cytoprotective effects in cardiomyocytes [61]. NF-*κ*B up-regulates also the Inhibitor of Apoptosis Proteins, X-linked Inhibitor of Apoptosis Proteins, and anti-apoptotic factors Bcl2 and cFLIP and indirectly prevents mitochondrial-mediated apoptosis by induction of Mn^2+^-superoxide dismutase (an ROS neutralizer) [62–64]. These data are in concordance with the fact that RelA and IKK knockout mice die from extensive liver apoptosis [40]. 

In DC, we found an increase of apoptosis and pro-apoptotic factors in the chronic diabetic rat heart [35]. Interestingly, NF-*κ*B was not activated at these stages of the disease. Other authors found similar results in early DC models [23, 41]. Activation of caspase cascade may be in part responsible for NF-*κ*B abrogation. In this sense, caspase-8 degrades RIP-kinase, a mediator needed for TNF-induced NF-*κ*B activation [3]. Thus, in contrast to the other events, activation of NF-*κ*B may be appropriate to attenuate the DC-associated apoptosis.

## 5. Potential NF-*κ*B-Based Therapeutic Approaches for DC

Since NF-*κ*B plays critical roles in the pathophysiology of several process of DC, it is plausible that exogenous modulation of NF-*κ*B activation may be effective to develop new therapeutic strategies. However, due to NF-*κ*B ubiquity, systemic NF-*κ*B inhibitors are likely to have deleterious side effects, particularly if used for long periods. When the original or ongoing stimuli for NF-*κ*B activation cannot be fully controlled, it is still possible to decrease their effects by interfering with the associated pathways. NF-*κ*B could be retained inactive in the cytoplasm by the use of drugs or genetic manipulations in the cardiac cell. Treatment with specific inhibitors against the proteasome degradation of I*κ*B has shown favourable effects in preventing NF-*κ*B activation [65]. Downstream, by using immunosuppressive agents such as cyclosporine-A, I*κ*B degradation is attenuated and NF-*κ*B remains also sequestered in the cytosol [66]. Also, the therapies with anti-oxidants like pyrrolidine dithiocarbamate, vitamins C or E, or ATP competitors as aspirin have been extensively used in heart injury research [67]. Further, once active NF-*κ*B enters into nucleus, it would be still feasible to modulate its transcriptional activity by lacking any interactive coactivator (i.e., other transcription factors). NF-*κ*B repression by glucocorticoid receptors (steroids treatment) has been successfully assayed [11]. Also, genetic delivery of I*κ*B*α* and genetic manipulations with small interfering RNA, oligodeoxynucleotides or dominant negative against IKK subunits, may be promising strategies for NF-*κ*B lessening in DC [8]. Lastly, some apparently NF-*κ*B-unrelated agents show a potential modulation of the NF-*κ*B pathway. For instance, angiotensin receptor blockers, angiotensin converting enzyme inhibitors and statins, exhibit beneficial effects in cardiac diseases although neither hypertension nor hyperlipidemia are present, possibly by NF-*κ*B attenuation [68]. 

However, all of these are unselective attempts to block NF-*κ*B activation that may be hazardous. Moreover, most of the beneficial effects have been shown for inflammation, but is uncertain the improvement of NF-*κ*B blockers in other DC-linked pathologies such as fibrosis, hypertrophy or apoptosis. It would be crucial to describe each one of the multiple ways through which NF-*κ*B can become activated in the different responses to DC, and specific alterations may be blocked selectively with reduced side effects. In this regard, we could profit from the anti-apoptotic role of NF-*κ*B and develop strategies in which anti-apoptotic pathways can be triggered in cardiomyocytes that are on their way to apoptosis, like in DC. Also, a further knowledge of the composition of the NF-*κ*B dimers and NF-*κ*B interacting partners would help to select the appropriate blockade. In this regard, increasing PPAR proteins, such as the *α* and *β*/*δ* isoforms, could mitigate the NF-*κ*B actions in the diabetic heart.

## 6. Conclusions

Both classical and alternative pathways of NF-*κ*B activation may participate in the deleterious responses of the cardiac cells to DC. Oxidative stress, inflammation, endothelial dysfunction, fibrosis, hypertrophy and apoptosis seem to be “attractive” processes for NF-*κ*B gene regulation. Thus, NF-*κ*B or, preferentially, any of its specific related pathways may be of highly interest for pharmaceutical target. A local tissue delivery of inhibitors that target a specific subunit or cofactor of NF-*κ*B or selective pathway(s) that leads to its modulation may work in DC.

##  Acknowledgments

This paper is funded by Ministerio de Sanidad y Consumo (SAF 2007/63648, 2007/60896 and 2009/8367), CAM (S2006/GEN-0247), Instituto de Salud Carlos III (FIS PI050451, PS09/01405 and CP04/00060), European Network (HEALTH F2-2008-200647), EUROSALUD (EUS2008-03565), Red RECAVA (RD06/0014/0035), Fundación Española del Corazón, Sociedad Española de Arteriosclerosis, Fundación Mutua Madrileña, MSD and Pfizer.

## Figures and Tables

**Table 1:** Potential main stimuli and NF-*κ*B-related genes involved in DC. Different stimuli can activate NF-*κ*B signalling (left) and several targeted genes (right) may be controlled by NF-*κ*B in the diabetic heart. COX2: cyclooxygenase-2; Mn^2+^-SOD: manganese-superoxide dismutase; IFN*γ*: interferon-*γ*; RANTES: regulated upon activation normal T cell expressed and secreted. The rest of abbreviations are described along the paper.

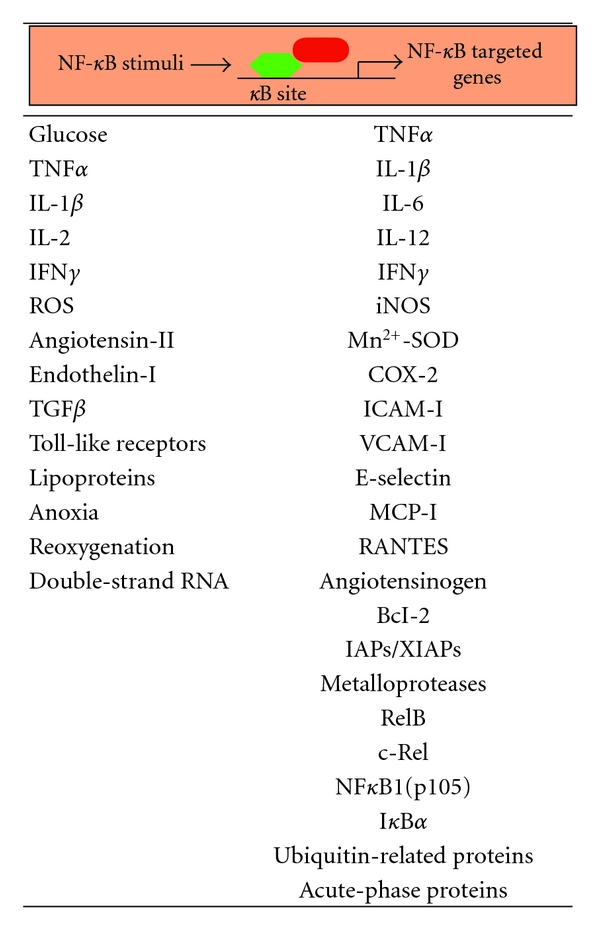

## Non-Standard Abbreviation and Acronyms

CREB: cAMP response element binding protein
STAT: Signal transducers and adaptors of transcription
NF-IL6: Nuclear factor IL-6
TNF*α*: Tumour necrosis factor-1*α*
IL: Interleukin
LDL: Low-density lipoprotein
VLDL: Very low-density lipoprotein
TRAF: TNF receptor-associated factors
TGF*β*: Transforming growth factor-*β*
CTGF: Connective tissue growth factor
RANK: Receptor activator of NF-*κ*B
NIK: NF-*κ*B inducing kinase
PKC: Protein kinase C
MCP-1: Monocyte chemoattractant protein-1
ICAM-1: Intercellular adhesion molecule-1
VCAM-1: Vascular cell adhesion molecule-1
PAI-1: Plasminogen activator inhibitor-1
ANP: Atrial natriuretic peptide
PGC-1*α*: PPAR*γ* coactivator-1*α*
Nkip1: NF-*κ*B interacting protein-1
IAPs: Inhibitor of apoptosis proteins
XIAPs: X-linked inhibitor of apoptosis proteins.
